# Supplementary material for: Dynamic expression of brain functional systems disclosed by fine-scale analysis of edge time series
Source: Netw Neurosci. 2021 Apr 27;5(2):405–33. doi: 10.1162/netn_a_00182 (PMC8233118; doi:10.1162/netn_a_00182)
Supplement: Supplementary file 1 [file netn-05-405-s001.pdf]

SUPPLEMENTARY INFORMATION

Dynamic Expression of Brain Functional Systems Disclosed by Fine-Scale Analysis of Edge Time Series

Olaf Sporns[1,2,3,4], Joshua Faskowitz[1,2], Andreia Sofia Teixeira[3,5,6], Sarah A. Cutts[1,2], Richard F. Betzel[1,2,3,4]

[1]Department of Psychological and Brain Sciences, Indiana University, Bloomington, USA.

[2]Program in Neuroscience, Indiana University, Bloomington, USA.

[3]Network Science Institute, Indiana University, Bloomington, USA.

[4]Cognitive Science Program, Indiana University, Bloomington, USA

[5] Center for Social and Biomedical Complexity, School of Informatics, Computing, & Engineering, Indiana University, Bloomington IN, USA

[6] INESC-ID, Lisboa, Portugal

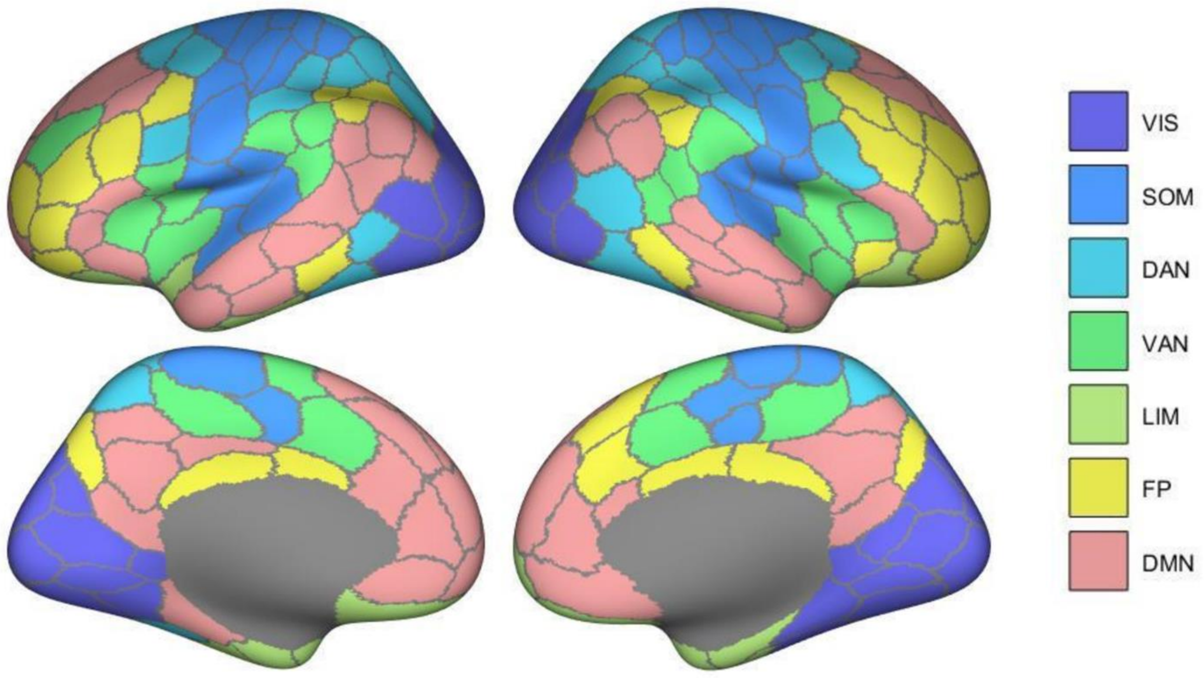

Figure S1: Canonical functional systems. Each of the 200 parcels was assigned to one of seven canonical resting-state networks (Yeo et al. 2011), comprising the visual (VIS), somatomotor (SOM), dorsal attention (DAN), ventral attention (VAN), limbic (LIM), frontoparietal (FP) and default mode (DMN) systems (Schaefer et al. 2018).

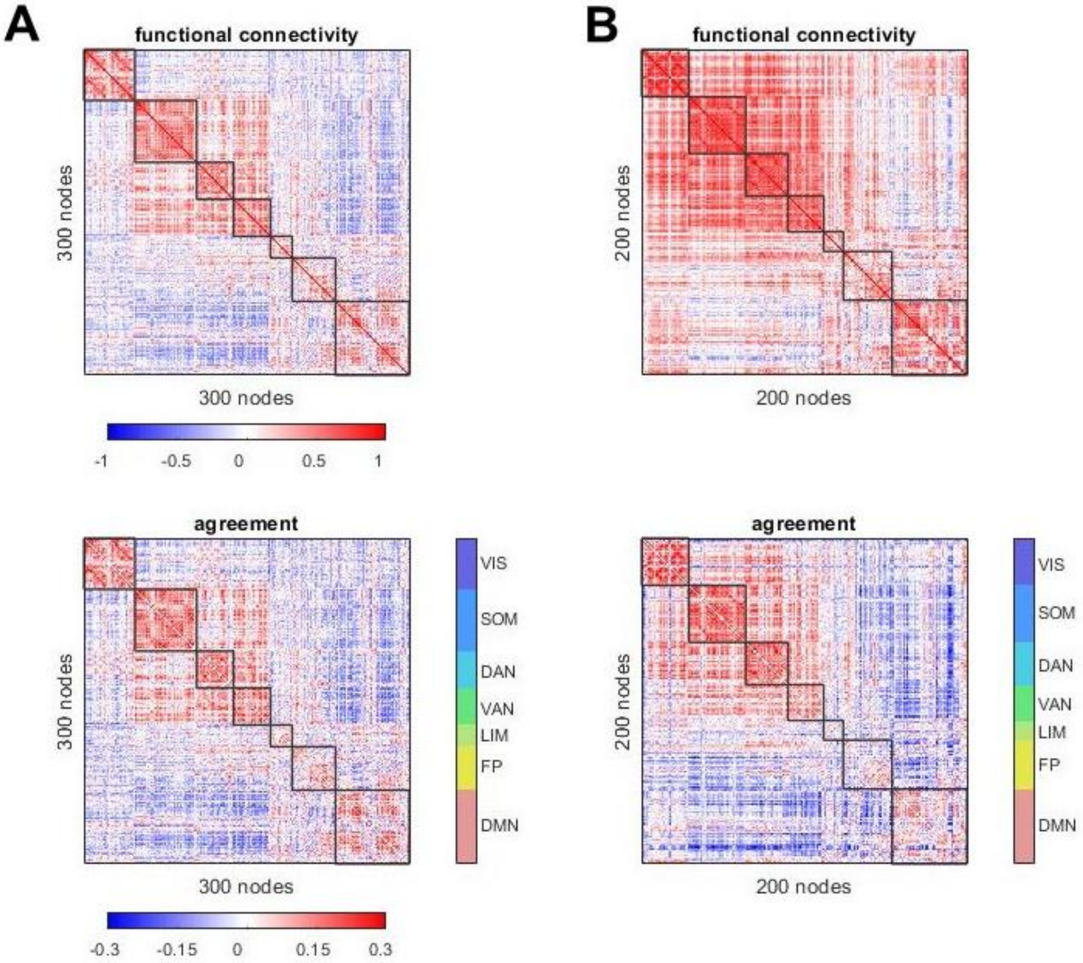

Figure S2: Comparison of FC and agreement matrix, for one representative participant, one imaging run, under finer (300 node) parcellation (A) and when omitting global signal regression from fMRI preprocessing (B). Across all 95 participants the corresponding mean correlations between FC and agreement are ρ = 0.960 ± 0.008 and ρ = 0.964 ± 0.009, respectively.

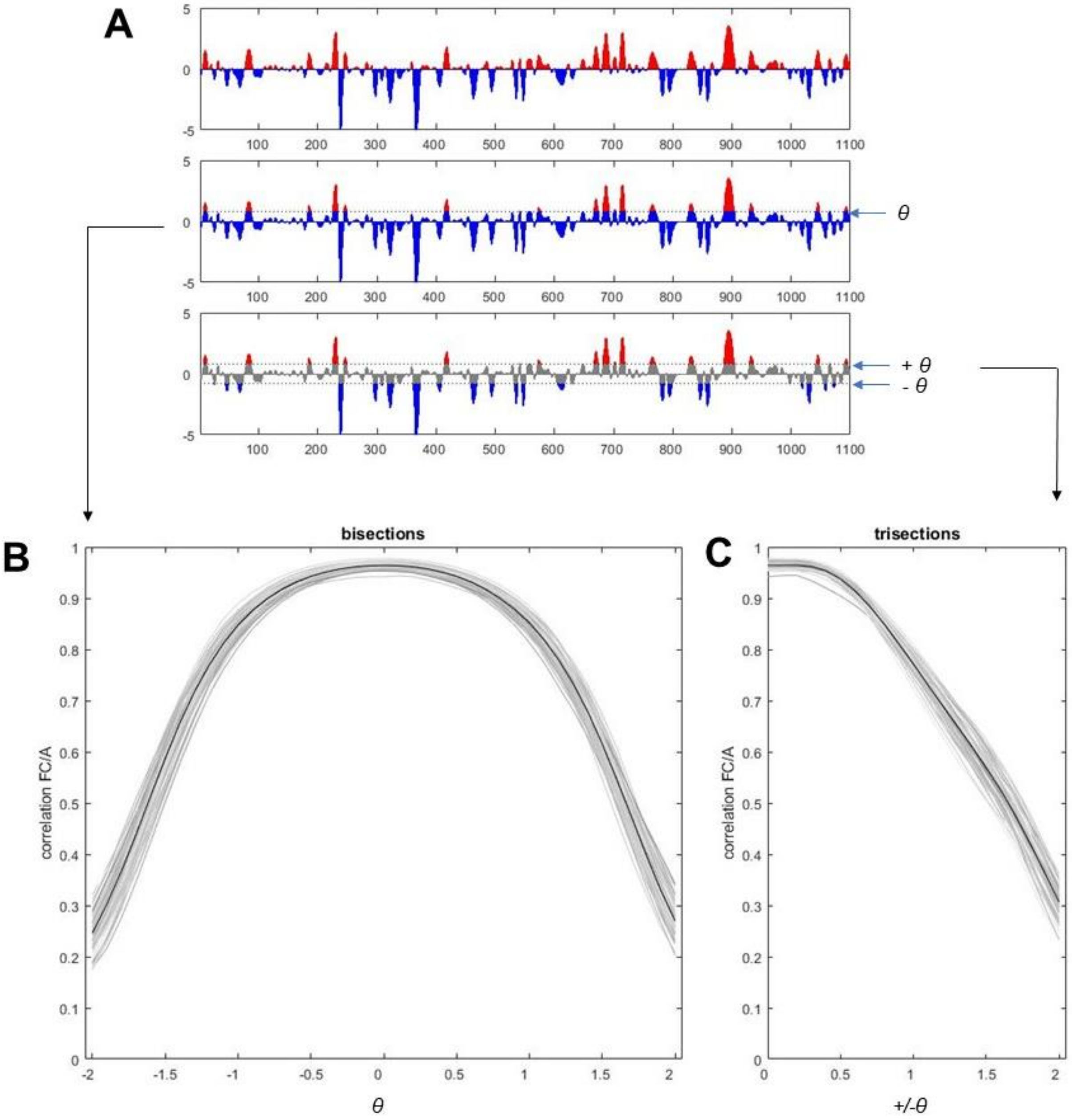

Figure S3: Variant for thresholding to determine bipartitions or tripartitions (A) and impact on correlation between FC and agreement matrices (B,C). (A) Plot at top shows example edge time series (cf. Fig 1) with a threshold applied at $z = 0$. Middle panel illustrates choice of an arbitrary threshold 'θ' (here θ = 0.8). Bottom panel shows application of two thresholds $+\theta$ and $-\theta$ to divide the time series into three bins. (B) Correlation (Spearman's ρ) as a function of parameter θ, for 95 participants (plot shows individuals as well as group mean). Note that the correlation remains strong over a wide range of the 'θ' parameter. (C) Same as panel B, but for tripartitions.

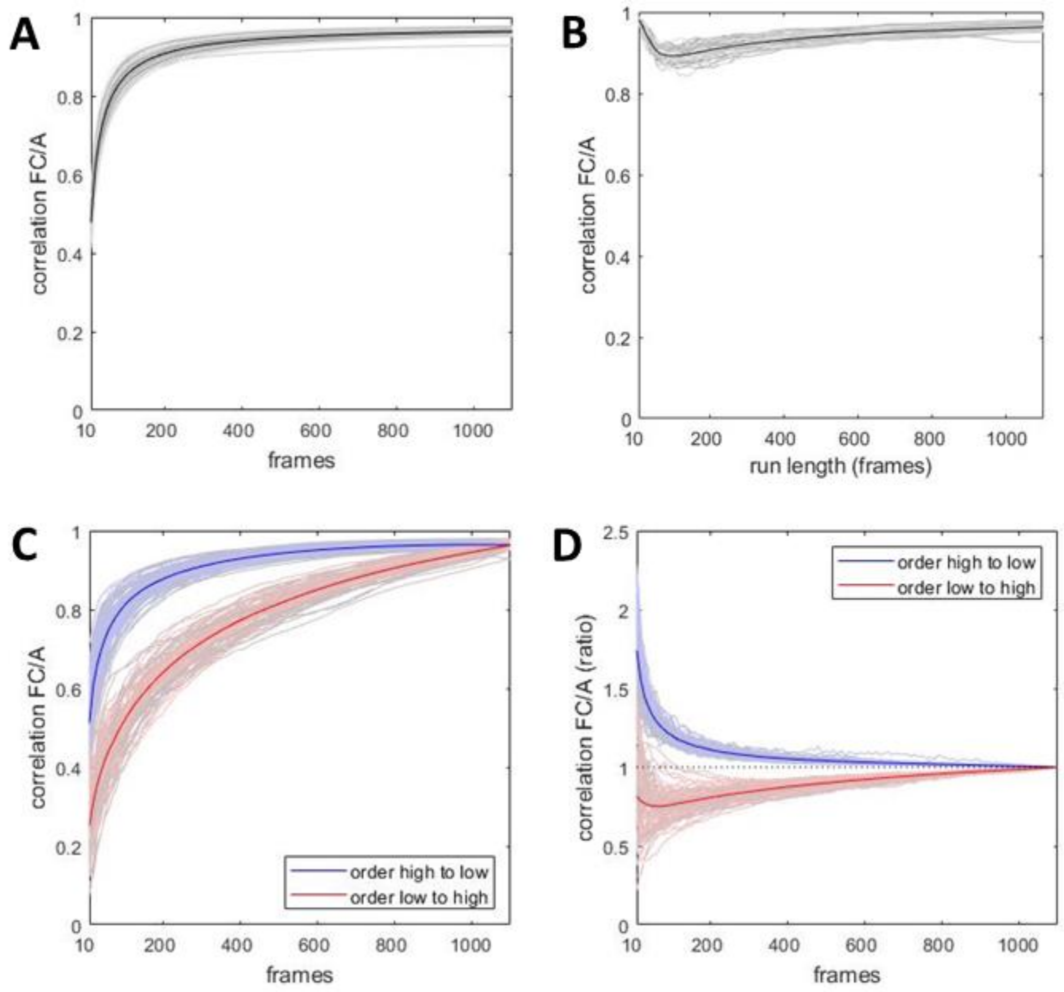

Figure S4: Approximation of FC by the agreement matrix. (A) Reconstruction of FC from bipartitions as a function of the number of randomly selected time steps (frames). The sample size is varied between 10 and 1100 frames (full length of imaging run), in steps of 10 frames. Correlation of agreement and (full-length) FC is plotted for each of 95 participants (light lines; means shown in black line). (B) Correlation between FC and agreement matrix as run length is varied from 10 to 1100 frames. FC and agreement matrices were computed for each run length, and thus are both derived from the same length time series data. Even short runs exhibit strong correlations between FC and the corresponding agreement matrix. (C) Same approach as in panel (A) but with frames selected after ordering them by RSS amplitude. Blue lines show results after selecting between 10 and 1100 frames in descending order of amplitude (going from high to low-amplitude frames), red lines show results after moving in reverse order (going from low to high amplitude frames). (D) Ratio of FC/A correlation when comparing data in panel (B) against a null model (25 independent runs) where frame numbers are shifted by a random offset, thus preserving their number, temporal spacing and hence signal autocorrelations. Ratios greater than 1 indicate better reconstruction than achieved by the null model.

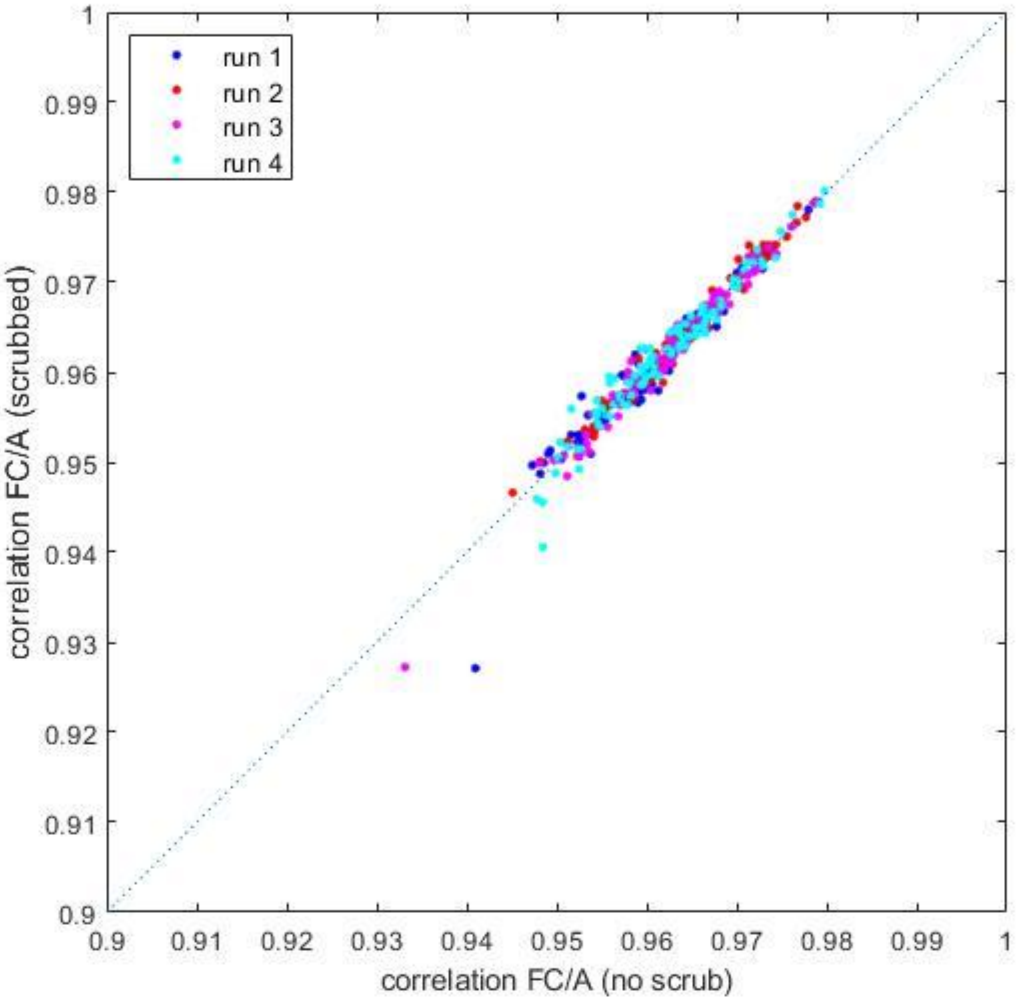

Figure S5: Impact of removing ('scrubbing') high-motion frames from each imaging run on the measured correlation between FC and agreement matrix. Scrubbing was carried out by removing all frames for which framewise displacement exceeded the 90th percentile for a given run. The agreement matrix of the remaining 90% of frames was compared (correlated) with the FC (all frames), plotted on the y-axis. The x-axis records correlations of agreement with FC when 90% of frames are sampled at random from the original time series (mean of 100 samples). The plots show data for all 95 participants and for all 4 imaging runs.

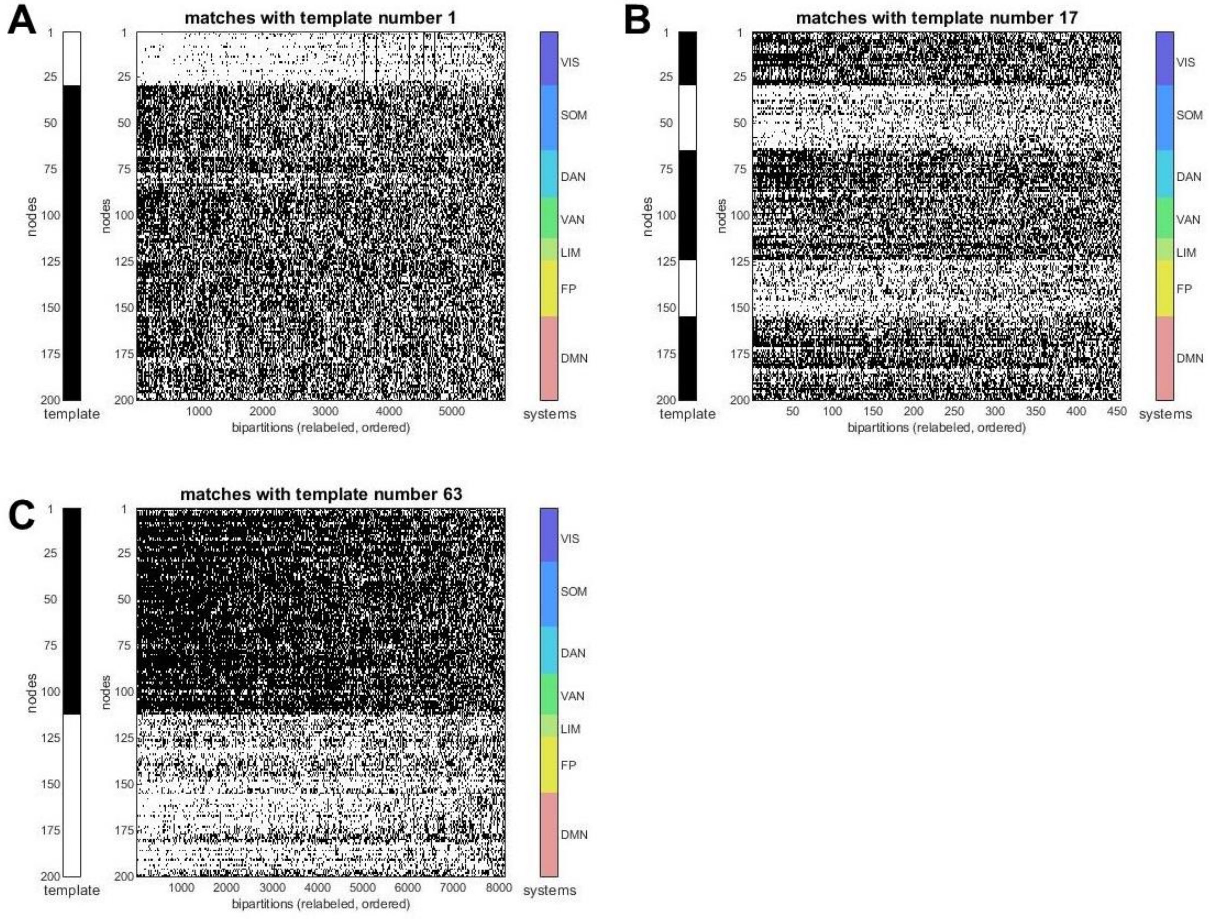

Figure S6: Examples of empirically observed bipartitions that were matched to three different target templates (templates 1, 17, 63; cf. Fig 5A). Bipartitions have been rectified to facilitate comparison to template vectors (shown to the left in each of the three panels).

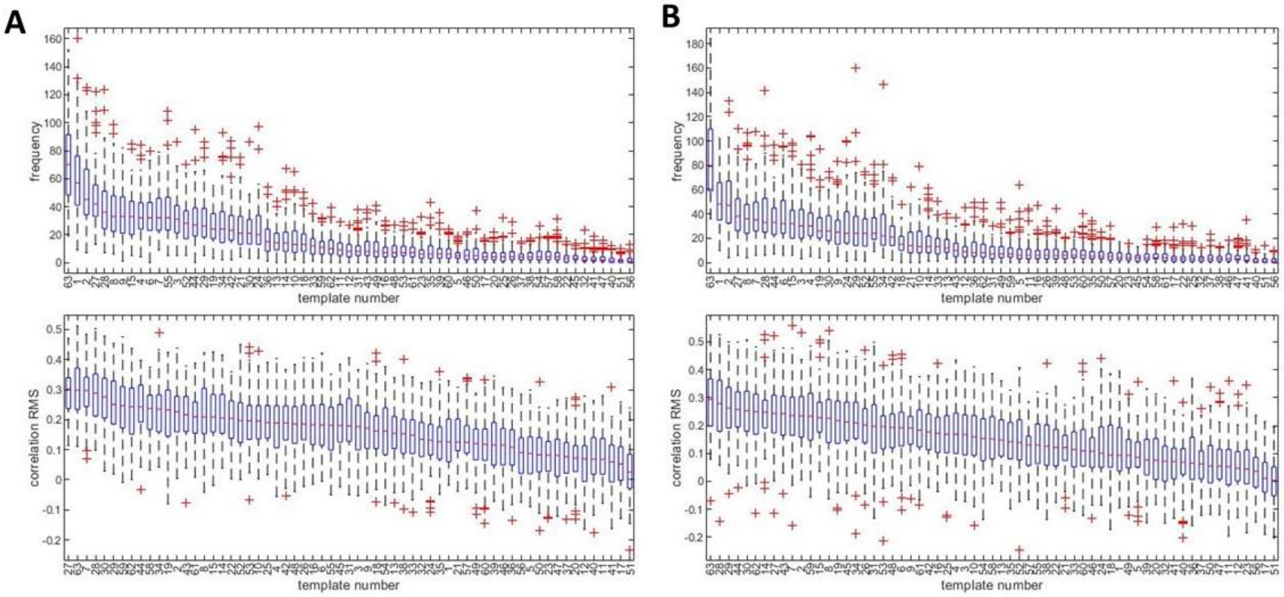

Figure S7: Template frequency and correlation with RSS, for data applying a finer (300 node) parcellation (A) and omitting global signal regression (B). Compare to Fig 5C,D in the main text.

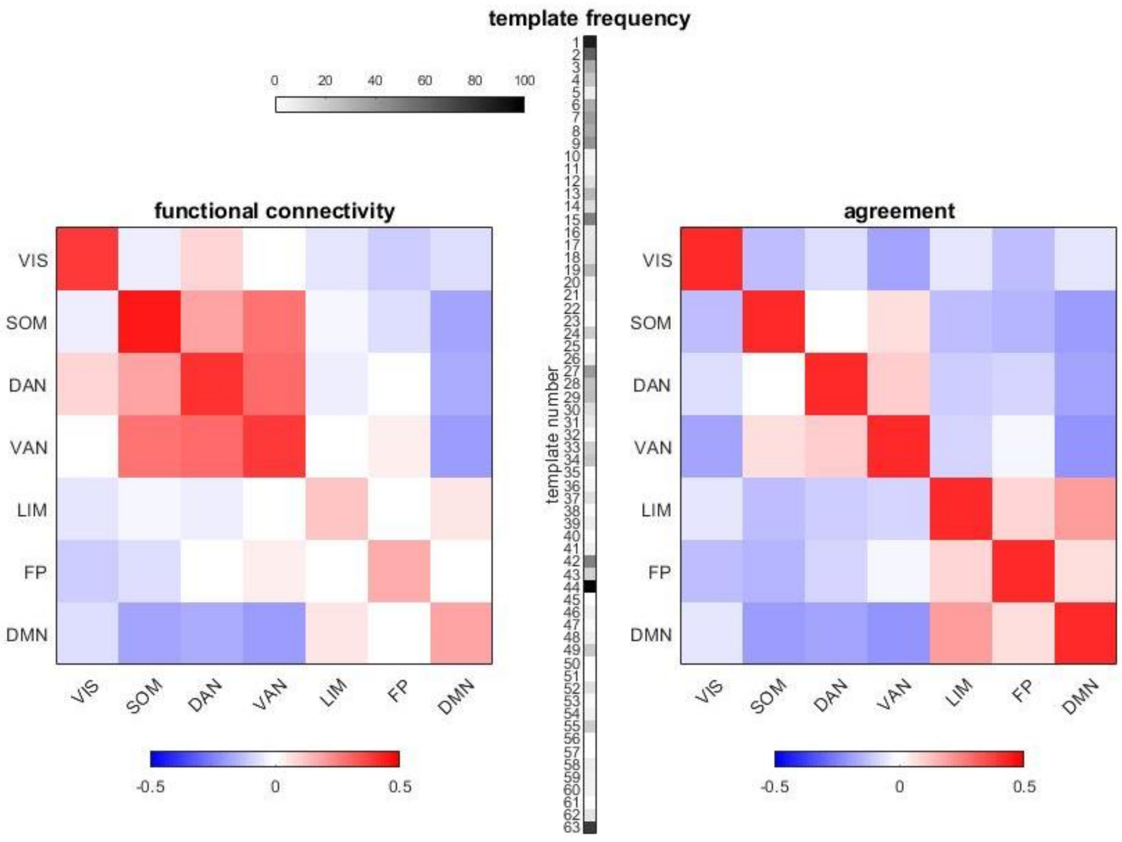

Figure S8: Down-sampled FC (7x7 matrix of functional systems), template frequency vector, and reconstructed agreement matrix, for one representative participant, single run. The template frequency is derived by comparing members of the template basis set to the observed bipartition on each time frame (cf. Fig 5). The agreement matrix on the right is computed from the agreement of the templates as encoded in the vector (middle). Note that structure within systems (matrix elements on the main diagonal) cannot be resolved due to the spatial resolution of the template set. The template frequency vector, which is a highly compressed representation of the original time series, reproduces significant variance in between-system interactions (compare to the down-sampled FC shown at the right).

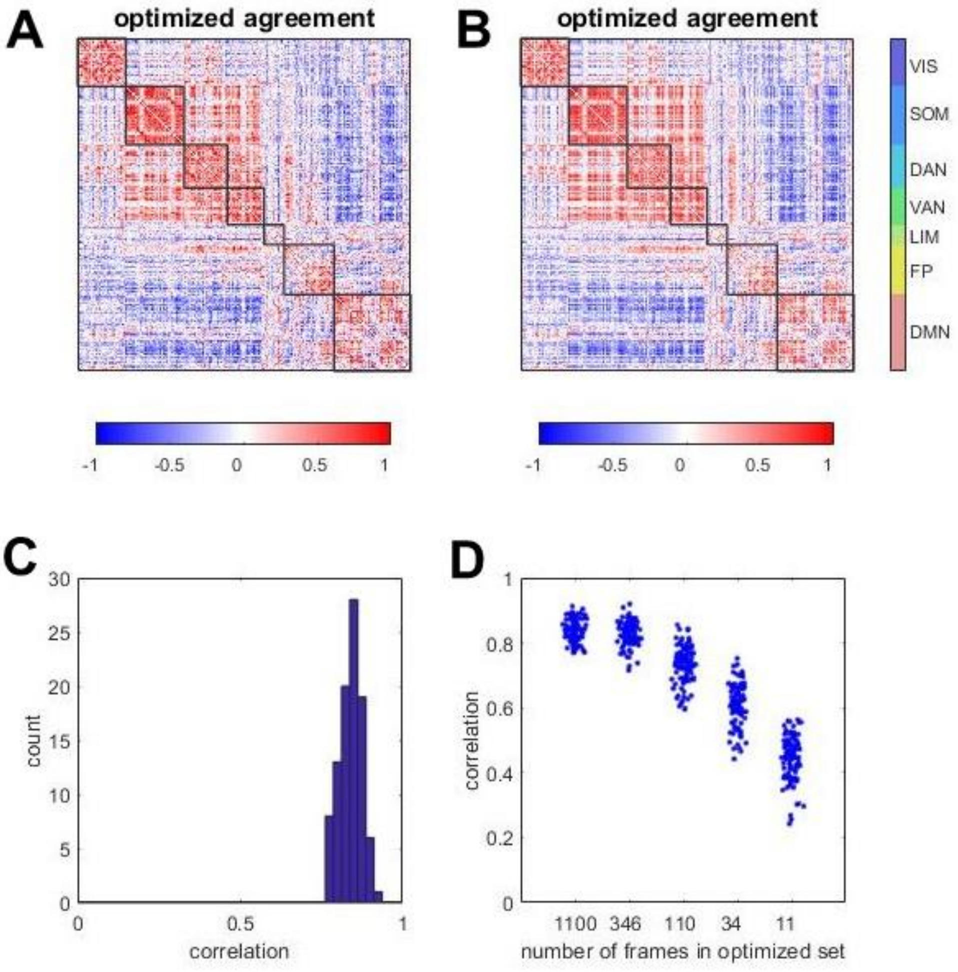

Figure S9: Data from optimizations using Spearman's ρ (A) or 'root-mean-square' (B) in the objective function. Both panels show optimized agreement matrices for the same participant shown in Fig 7. (C) Histogram of match between template sets in observed and optimized bipartitions, over all 95 participants, single run. (D) Match between template sets in relation to the size (number of bipartitions) of the optimized set. Note that even much more compact sets yield bipartition frequencies that closely match those observed across all 1100 frames in the original time series. In all cases, optimized templates are significantly similar to those found in the observed set.

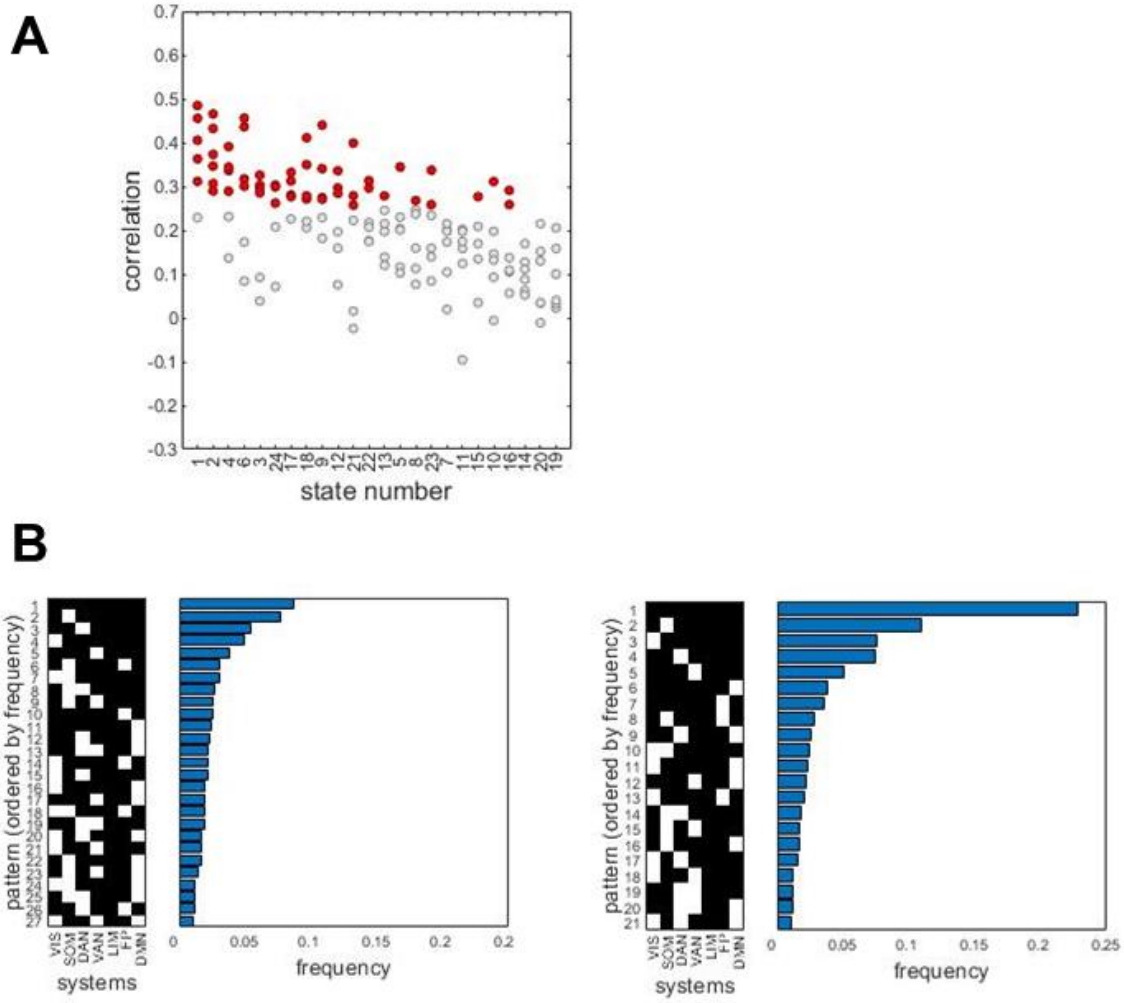

Figure S10: (A) Correlations of frequencies of system states, computed over 95 participants across pairs of 2 runs (6 pairs of runs). Correlations were compared against 10,000 random shuffles of participant labels, with data points in red corresponding to instances when the empirical correlation exceeded the null distribution with $p < 0.05/6$. (B) System states expressed during more than 1% of all frames, ranked by their frequency, when the z-threshold is varied ($z = 5$, left panel; $z = 8$, right panel).
